# Exonization of an Intronic LINE-1 Element Causing Becker Muscular Dystrophy as a Novel Mutational Mechanism in Dystrophin Gene

**DOI:** 10.3390/genes8100253

**Published:** 2017-10-03

**Authors:** Ana Gonçalves, Jorge Oliveira, Teresa Coelho, Ricardo Taipa, Manuel Melo-Pires, Mário Sousa, Rosário Santos

**Affiliations:** 1 Unidade de Genética Molecular, Centro de Genética Médica Dr. Jacinto Magalhães, Centro Hospitalar do Porto, 4050-106 Porto, Portugal; ana.goncalves@chporto.min-saude.pt; 2 Unidade Multidisciplinar de Investigação Biomédica (UMIB), Instituto de Ciências Biomédicas Abel Salazar (ICBAS), Universidade do Porto, 4050-313 Porto, Portugal; msousa@icbas.up.pt; 3 Serviço de Neurofisiologia, Departamento de Neurociências, Centro Hospitalar do Porto, 4099-001 Porto, Portugal; tcoelho@netcabo.pt; 4 Unidade de Neuropatologia, Centro Hospitalar do Porto, 4099-001 Porto, Portugal; ricardotaipa@gmail.com (R.T.); melopires@hotmail.com (M.M.-P.); 5 Departamento de Microscopia, Laboratório de Biologia Celular, Instituto de Ciências Biomédicas Abel Salazar (ICBAS), Universidade do Porto, 4050-313 Porto, Portugal; 6 Centro de Genética da Reprodução Prof. Alberto Barros, 4050-313 Porto, Portugal; 7 UCIBIO/REQUIMTE, Departamento de Ciências Biológicas, Laboratório de Bioquímica, Faculdade de Farmácia, Universidade do Porto, 4050-313 Porto, Portugal

**Keywords:** Becker muscular dystrophy, cDNA, *DMD*, Dystrophin, LINE-1

## Abstract

A broad mutational spectrum in the dystrophin (*DMD*) gene, from large deletions/duplications to point mutations, causes Duchenne/Becker muscular dystrophy (D/BMD). Comprehensive genotyping is particularly relevant considering the mutation-centered therapies for dystrophinopathies. We report the genetic characterization of a patient with disease onset at age 13 years, elevated creatine kinase levels and reduced dystrophin labeling, where multiplex-ligation probe amplification (MLPA) and genomic sequencing failed to detect pathogenic variants. Bioinformatic, transcriptomic (real time PCR, RT-PCR), and genomic approaches (Southern blot, long-range PCR, and single molecule real-time sequencing) were used to characterize the mutation. An aberrant transcript was identified, containing a 103-nucleotide insertion between exons 51 and 52, with no similarity with the *DMD* gene. This corresponded to the partial exonization of a long interspersed nuclear element (LINE-1), disrupting the open reading frame. Further characterization identified a complete LINE-1 (~6 kb with typical hallmarks) deeply inserted in intron 51. Haplotyping and segregation analysis demonstrated that the mutation had a de novo origin. Besides underscoring the importance of mRNA studies in genetically unsolved cases, this is the first report of a disease-causing fully intronic LINE-1 element in *DMD*, adding to the diversity of mutational events that give rise to D/BMD.

## 1. Introduction

Duchenne or Becker muscular dystrophies (D/BMD), caused by pathogenic variants in the Dystrophin (*DMD*) gene, are among the most common inherited diseases of muscle, with an estimated prevalence of ~1/3800 live male births [1]. A broad mutational spectrum for D/BMD has been thoroughly described in the literature, ranging from large multi-exonic deletions/duplications to smaller single nucleotide variants [2]. More complex and rarer *DMD* mutations, such as large rearrangements and gene disruption mediated by retrotransposition activity, have also been reported [3,4]. The genetic heterogeneity, size, and complexity of the *DMD* gene demands expertise in a vast number of molecular techniques, besides the routinely used multiplex-ligation probe amplification (MLPA) and genomic sequencing. Since dystrophinopathies are now amenable to therapy, the genetic characterization of these patients has gained relevance beyond clinical follow-up and genetic counselling purposes.

We previously reported the characterization of 308 dystrophinopathy patients, from 284 unrelated families, leading to the identification of 175 distinct mutations [5]. This 91% positivity rate (284 of 312 families) was achieved in a cohort with strict inclusion criteria. Since then, and considering all referrals with clinical suspicion of D/BMD, over 100 cases remain unsolved at the genetic level.

This report describes a wide combination of genetic studies performed on a patient presenting a mild BMD phenotype, where a unique mutational event involving the insertion of a long interspersed nuclear element 1 (LINE-1) was identified.

## 2. Materials and Methods

### 2.1. Patient Samples

Formal written informed consent for publication of this case report was obtained from the patient and other family members whose data is presented. The study was conducted in accordance with the Declaration of Helsinki, and with approval of the institutional (CHP) ethics committee (Code: 336-13(196-DEFI/285-CES); date of approval: 11 December 2013).

### 2.2. RNA Studies

Total RNA extracted from patient and control muscle samples with PerfectPure RNA Fibrous Tissue kit (5 PRIME) was converted to cDNA using the High Capacity RNA-to-cDNA Kit (Thermo Fisher Scientific, Waltham, MA, USA). *DMD* transcripts were amplified by PCR covering the entire coding region. Amplicons were purified with Illustra ExoStar (GE Healthcare, Little Chalfont, UK) and sequenced using BigDye^TM^ Terminator Cycle Sequencing Kit V3.1 (Thermo Fisher Scientific). Reference sequence for variant description: NM_004006.2.

### 2.3. Bioinformatics

Genome similarity sequence search was conducted using the Basic Local Alignment Search Tool (BLASTN v2.2.32) [6]. Analysis of repetitive elements was performed using CENSOR [7], RepeatMasker [8] and L1Xplorer [9]. Potential acceptor splice-sites and branchpoints were assessed using different algorithms available in Alamut Visual software (v2.8, Interactive Biosoftware, Rouen, France) (Supplementary Materials Figure S1).

### 2.4. LINE-1 Characterization

In order to identify the 5′ insertion site, primers were designed against five candidate target regions within intron 51 (Supplementary Materials Data S1). For the 3′ insertion site, a forward primer was designed against a conserved region of the LINE-1 3′UTR (L1-F: AAATTAGGTATTGATGGGACGTATT) and a reverse primer within intron 51 (51int-R: GAGAAGATGACAGTTAAATCAAAGC) (Supplementary Materials Data S1). Resultant amplicons were sequenced as described above. LINE-1 was genotyped by single molecule real-time sequencing (PacBio RS II system, Pacific Biosciences, San Francisco, CA, USA) using custom DNA libraries (Supplementary Materials Data S1). FASTA/Q files were mapped against a LINE-1 reference and consensus sequence was obtained from BAM files using Samtools mpileup command (Supplementary Materials Data S1). Sequence artifacts and ambiguous sites were clarified via long-range PCR followed by Sanger sequencing.

## 3. Results

**Figure 1:**
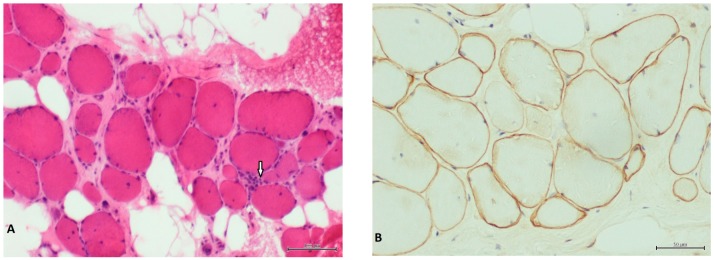
Patient’s muscle biopsy. (**A**) Hematoxylin and eosin stain showing severe fibrosis and fat substitution. Arrow indicates a necrotic fiber. Scale bar corresponds to 100 µm. (**B**) Dys2 antibody showing irregular and faint staining of dystrophin. Scale bar corresponds to 50 µm.

We report the genetic characterization a 50-year-old male patient with clinical features compatible with BMD, namely, onset at 13 years of age with progressive proximal weakness of lower limbs, electromyography showing myopathic signs, and high creatine kinase levels. The patient was initially referred for *DMD* molecular testing in 2001, but multiplex PCR and Southern blot failed to detect pathogenic variants. The case was re-evaluated in the context of genetic counselling of the patient’s daughter. A muscle biopsy performed in the patient revealed dystrophic features and irregular staining for Dystrophin (Figure 1).

**Figure 2:**
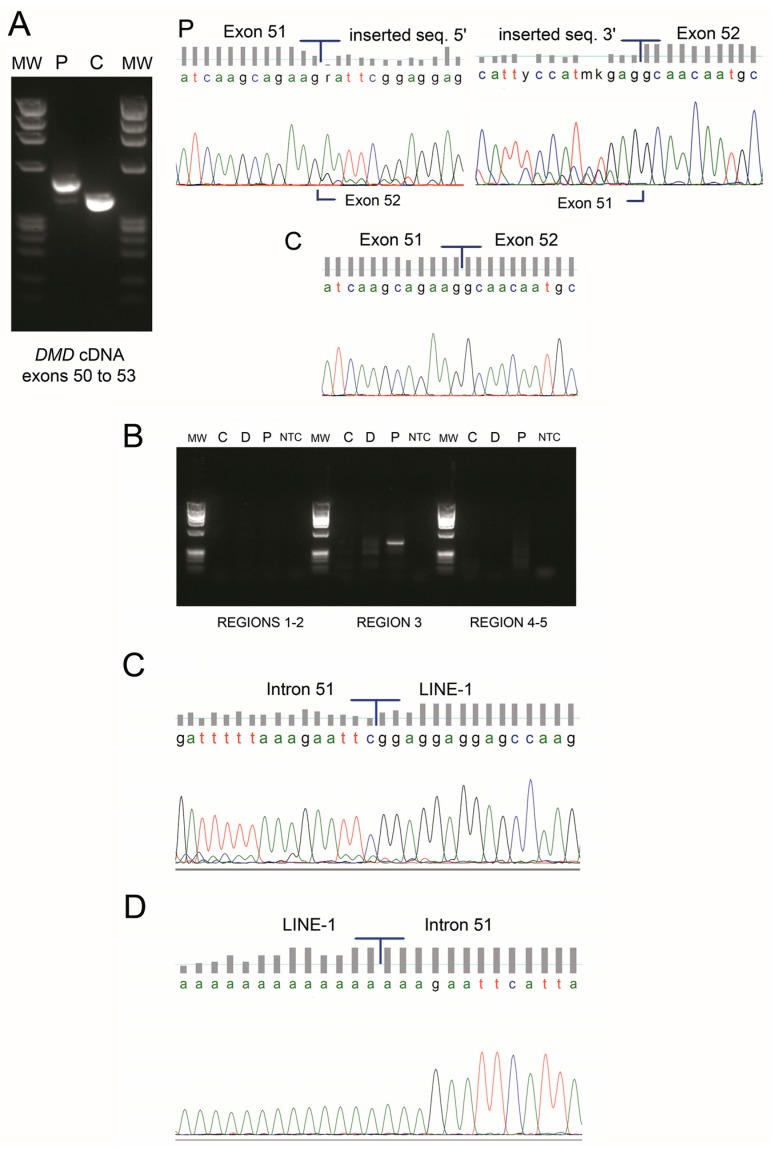
(**A**) cDNA analysis of *DMD* transcript revealed an abnormal PCR product with higher molecular weight in the patient (P) which was not detectable in a control sample (C). Sequencing electropherogram shows an insertion of 103 nucleotides between exons 51 and 52. A residual wild-type transcript is present in the patient sample (faint PCR band). (**B**) PCR amplification of five candidate regions to identify the long interspersed nuclear element (LINE-1) insertion site in intron 51 of *DMD* (C—control, D—control with a deletion of intron 51, P—patient, MW—molecular weight marker, NTC—no template control). A single amplicon was obtained in the patient sample for the candidate region 3. (**C**) Upon sequencing, the exact location of the LINE-1 in intron 51 was mapped to lie between positions c.7542+8951 and c.7542+8952 (NM_004006.2). (**D**) The 3′ insertion site was confirmed through a specific PCR followed by sequencing; the LINE-1 poly-A tail is also seen.

Given that large deletions/duplications and point mutations were not detected by current routine genetic studies (MLPA and *DMD* genomic sequencing), complete *DMD* cDNA analysis of the muscle specimen was performed. An insertion of 103 nucleotides (not traceable in *DMD*) was identified between exons 51 and 52 (r.7542_7543ins(103), Figure 2A), predictably shifting the *DMD* open reading frame (ORF). Besides the predominant mutated transcript, a residual amount of wild-type transcript was detectable (Figure 2A). To identify the origin of the mutated sequence, a BLASTN query was performed against the human nucleotide collection. Identity of 95.1% (98/103 base pairs (bp)) was retrieved against two human LINE-1 sequences: L1.21 and L1.14 (GenBank accession numbers U93570 and U93566, respectively) (Supplementary Materials Figure S2). Comparative analysis also showed high similarity with other human genomic sequences containing LINE-1 elements. Further confirmation was obtained using CENSOR software where 98/103 bp had 100% similarity with the consensus sequence of the human LINE-1 element (Supplementary Materials Figure S2).

A strategy was delineated to identify the genomic insertion site in the *DMD* gene. The first five nucleotides (AATTC), having no correspondence to the LINE-1 consensus sequence, were presumed to belong to intron 51 of *DMD*. This ~44 Kb intron was scanned for the sequence AG/AATTC (AG being the canonical dinucleotide for acceptor splice-sites); a total of eight such sequences were found. Composite splice-site analysis narrowed this down to five potential sites—those presenting high splice-site scores and suitable (cryptic) branch-points located nearby (Supplementary Materials Figure S1). To identify the LINE-1 5′ insertion site by PCR, three forward oligonucleotides were designed to encompass these five regions of interest and a single reverse oligonucleotide annealing to the known inserted LINE-1 sequence (detected by cDNA analysis). PCR experiments and subsequent sequencing showed that the LINE-1 was inserted at position NM_004006.2:c.7542+8951_c.7542+8952 of intron 51 (Figure 2B,C). The 3′ end was then identified, containing a poly-A tail and a stretch of 9 bp (AAAGAATTC) consistent with a flanking target site duplication (TSD) (Figure 2D). Southern blot and hybridization was performed to estimate the size of insertion. Results revealed a ~6 Kb size increase, thus corresponding to a complete or almost complete LINE-1 element (Supplementary Materials Figure S3).

**Figure 3:**
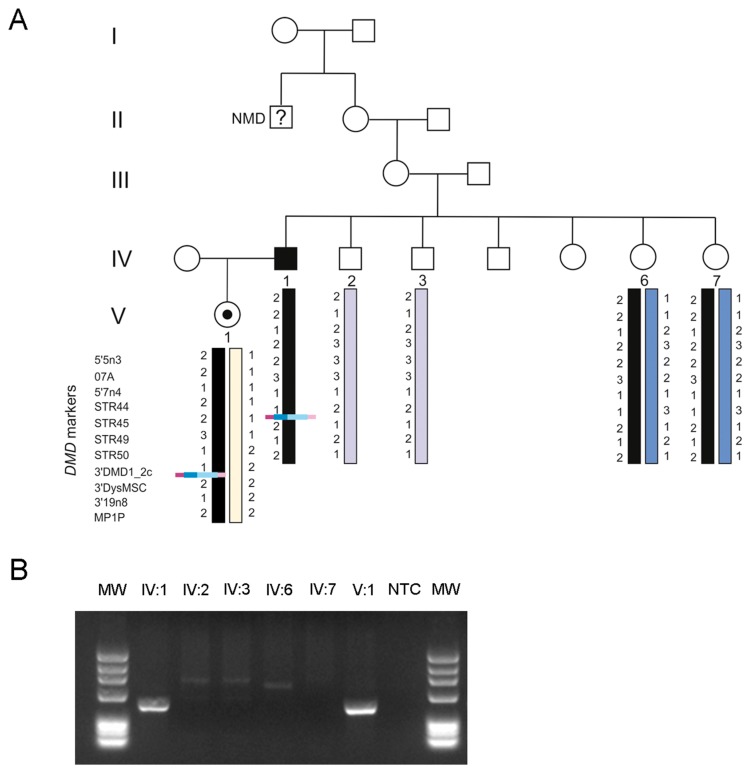
(**A**) Patient’s family tree highlighting the segregation of the LINE-1 insertion and *DMD* haplotyping. This insertion is present in the patient (IV:1) and his daughter (V:1), and not detectable in the patient’s sisters (IV:6 and IV:7 both carriers of the same at-risk haplotype). Interestingly, this family was initially thought to have an X-linked transmission, since one of the patient’s maternal great-uncles (deceased) was suspected to have a neuromuscular disease (NMD). (**B**) LINE-1 specific PCR used to screen for additional carriers.

The patient’s daughter was seen to be a carrier of the LINE-1 insertion mutation. Additional family members (the patient’s healthy brother and two sisters presenting at-risk haplotypes) were also screened for the mutation using a LINE-1-specific PCR (Figure 3). Only the patient´s daughter tested positive, suggesting a de novo event.

**Figure 4:**
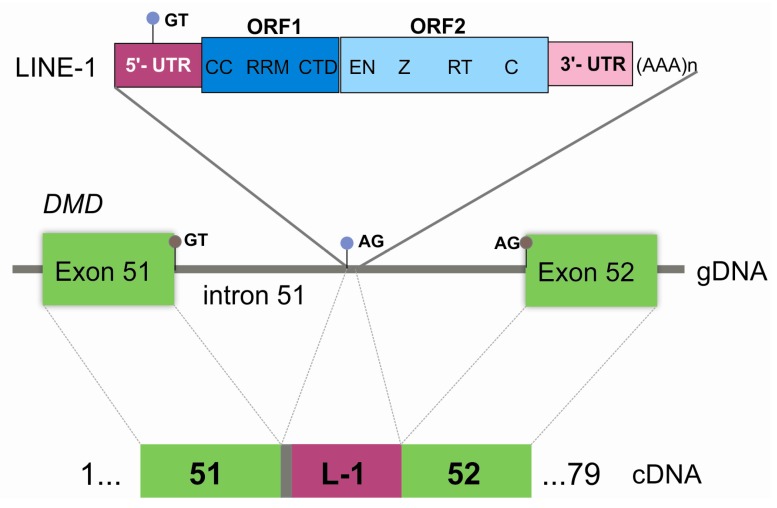
Graphical representation of the pathogenic LINE-1 insertion. At the genomic level (gDNA), the integration site is located in intron 51 of *DMD*. LINE-1 is displayed showing its characteristic features: 5′UTR, open reading frame (ORF) 1 (CC—coiled coiled domain, RRM—RNA recognition motif, CTD—C-terminal domain), ORF2 (EN—endonuclease, Z domain, RT—reverse transcriptase, C—cysteine-rich), and 3′ UTR with a poly-A tail. At the cDNA level, the retention of five base pairs from intron 51 (grey box), and the partial exonization of LINE-1 (L-1) (dark pink box) is explainable by the recognition of cryptic splice-sites (light blue circles) located in intron 51 (acceptor splice-site) and in the LINE-1 sequence itself (donor splice-site). Gray circles correspond to the canonical splice-sites.

Further genotyping confirmed that a full-length LINE-1 was present (sequence available in Supplementary Materials Data S2 and submitted to GenBank, accession number MF421743). L1Xplorer and RepeatMasker tools classified the element as a member of the L1HS subfamily, as it had all the typical hallmarks of these retrotransposons: a 5′-untranslated region (UTR), two non-overlapping ORFs (ORF1 and ORF2), a short 3′UTR and a poly-A tail (Figure 4).

## 4. Discussion

LINE-1s are the most abundant type of retrotransposable elements, accounting for nearly 17% of the human genome [10]. Typically, they are ~6 kb in length and exhibit characteristic components (Figure 4). ORF1 encodes an RNA-binding protein while ORF2 encodes a protein with endonuclease and reverse transcriptase activity. Although the transcriptional mechanism of LINE-1 is not fully understood, it has been proposed to involve target-site primed reverse transcription. The cDNA originated by this process recombines with genomic DNA, giving rise to characteristic signatures: a 7–20 bp direct repeat of the endonuclease target flanking the inserted LINE-1 (TSD) [11]. Only 80–100 LINE-1s in the human genome (0.1% of total) are believed to be capable of active retrotransposition [11].

Despite their importance in evolution and genome diversity, the insertion of a LINE-1 within a gene could have a deleterious effect, giving rise to disease. To date, only 30 such insertions have been reported, the majority located in exonic regions and causing frame-shifts or exon skipping [11]. In contrast, intronic LINE-1 insertions are rarely reported.

Regarding the *DMD* gene, only five pathogenic insertions have been described. Exonic disruptions, giving rise to a DMD phenotype, have been reported twice in exon 44 [12,13], and also in exons 48 and 67 [14,15]. A further pathogenic insertion was detected in two unrelated Japanese families with X-linked dilated cardiomyopathy, where a 5′-truncated form of a LINE-1 was integrated in the *DMD* 5′UTR, thought to affect the transcription or the stability of muscle transcripts [16]. A different repetitive element (Alu-like) was also reported to cause dilated cardiomyopathy, activating a cryptic acceptor splicing site in intron 11 of *DMD* [17]. The mutational event in our patient is completely distinct in two aspects: it is a deep-intronic insertion and a full LINE-1 sequence is present. This LINE-1 was classified as a member of the L1HS subfamily, responsible for the majority of the documented LINE-1 retrotransposition events. Our results showed its partial exonization at the cDNA level, due to the recognition of a cryptic 3′ splice-site located in intron 51 and a 5′ splice-site within this element (Figure 4). This presumably gives rise to a truncated polypeptide (p.Ala2515Asnfs*21). The presence of a residual wild-type transcript explains the patient’s milder dystrophinopathy (BMD phenotype). This LINE-1 sequence has 100% identity with a LINE-1 element located in chromosome 2 (GenBank accession number AC216112), which could constitute its original source. It has a near-complete identity (only one bp difference) with another pathogenic LINE-1 (GenBank accession number AF149422) inserted in the hemoglobin-beta *locus*; a seemingly active retrotransposable element of the human genome [18].

Intronic LINE-1 insertions causing exonization have only been described in three other cases: chronic granulomatous disease (*CYBB* gene) [19], Chanarin-Dorfman syndrome (*ABHD5* gene) [20], and familial retinoblastoma (*RB1* gene) [21]. The rarity of LINE-1-mediated pathogenic insertions described in the literature and in variant databases is attributable mostly to their low activity throughout the genome and to the technical difficulties in their detection (especially the full-length insertions). With massive parallel sequencing technology, there are also considerable limitations, particularly through short reads sequencing, and here, tailored bioinformatics analysis tools and strategies are required [22]. In the case of intronic LINE-1 insertions, detection may be hampered by the intron’s length and the fact that it mainly affects transcriptional events (e.g., intronic retentions or exonization events). One possibility is therefore to conduct mRNA studies in yet uncharacterized B/DMD patients, as previously suggested [5,23].

It is known that repetitive transposable elements such as short interspersed nuclear elements (e.g., Alu sequences) or LINE sequences are frequent in *DMD* intronic regions. However, besides being the underlying cause of some gross rearrangements, they may also influence gene expression by mediating alternative splicing. One can thus speculate that this would ultimately interfere with the efficacy of RNA-based therapies, especially those designed to restore the reading frame. To our knowledge, this is the first report of a deep-intronic insertion of a LINE-1 element in the *DMD* gene shown to cause disease. Besides its scientific relevance, while expanding the mutational mechanisms underlying B/DMD, this finding also reinforces the need to develop comprehensive approaches to identify LINE-1 insertion profiles in the human genome.

## Supplementary Materials

The following are available online at www.mdpi.com/2073-4425/8/10/253/s1. Figure S1: Bioinformatic splice-site, and branch-point analysis, Figure S2: Homology search bioinformatics, Figure S3: Southern blot analysis, Data S1: Additional material and methods for the LINE-1 characterization, Data S2: LINE-1 sequence.
