# Supplementary material for: Exonization of an Intronic LINE-1 Element Causing Becker Muscular Dystrophy as a Novel Mutational Mechanism in Dystrophin Gene
Source: Genes (Basel). 2017 Oct 3;8(10):253. doi: 10.3390/genes8100253 (PMC5664103; doi:10.3390/genes8100253)
Supplement: Supplementary file 1 [file genes-08-00253-s001.zip › Data S1.pdf]

Data S1. Additional material and methods for the LINE-1 characterization.

I. Primers used to identify the LINE-1 5′ insertion site in *DMD*.

a) Schematic showing primers' location:

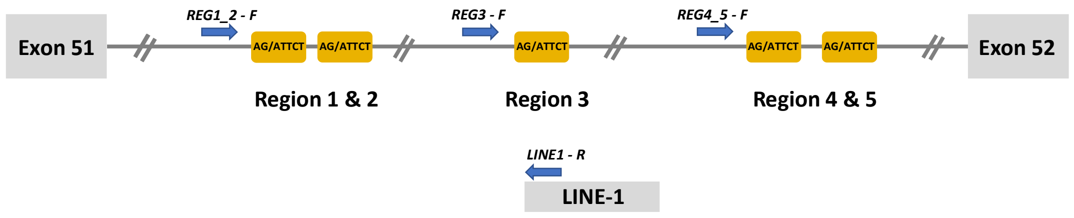

b) Primers sequences:

REG1_2-F: TCAAGCAAAACTGATCCAGG

REG3-F: GCAGTCATGGCCACAAAAG

REG4_5-F: ACAAAATCTATGGAGGCACAGG

LINE1-R: TGGAAATGCAGAAATCACCC

II. Sequences of custom probes used for isolating and enriching target region by hybridization capture of DNA libraries for (PacBio) single molecule real time sequencing.

> Probe nr1 (NM_004006.2:c.7542+8207_7542+8516)

AGATGAGGCAGTGATCACACATCACTATTAGTAAAAGTGTTTCTGTACCTGTAT
CCACACTTTTATGTATATGGTTACTTATGTTAAAGTGATACATATTATATAAAAT
TAACGTATACATTAAGTAGATATTTTAATAGTCTGTAATTAAATACTACTAGTAT
TTTCTTTCCTCCTTCAAGTGCTTACTTTTGATACCTCGAGTTACAGTGTCATAAAG
ATTCTTTAGAAATATATTGACTGTCTTTTAAGAGCTTTTGATACAATACTGAGTTT
ACATTCATCTGTTATTTATTGAACACTTGCTGGTGAAAGGCATC

Primers: F: AGATGAGGCAGTGATCACACA R: GATGCCTTTCACCAGCAAGT

> Probe nr2 (NM_004006.2:c.7542+9241_7542+9589)

AATGTAACCCCTCCCCCATATCAAGTTAATCTATGTTCAACTCCAGAATTATTTT
TGAACACTCAAACTAGAAATTAAAAAAAATTAAATCCATGAAGACGATTTTTG
CCAAAAGCATATAGATAAATTGAGTTGATTCTATACTTAAGAAAGTGGAGAGG
AGAGAGTAATTTGGAGAGAGTAATTTACTCTTAATCCCATATTTTTTCCCTAAAT
GTGAAAGAAGTAGATTGTAGTGAGAGGGAAAATAACCTGTAGCAACTTCATTG
AGGCTAAGCTTTCTGTCATGTTATATTATACGAAAGTAATGAAATGCTTCCACA
GATAGAATCAGAAGTCCCCTCTGAGA

PRIMERS: F: AATGTAACCCCTCCCCCATA R: TCTCAGAGGGGACTTCTGATTC

III. Custom bioinformatic pipeline for single molecule real-time sequencing analysis

a) FASTA/Q files were mapped against a LINE-1 reference sequence (ID: L1HS from http://www.girinst.org/repbase/) using BLASR alignment software ("-clipping soft -minMatch 7 -minPctSimilarity 90") (Chaisson et al., 2012).
b) BAM files were generated from SAM files with Samtools (v0.1.19; "view -b -S") (Li et al., 2009).
c) Consensus sequence was called from BAM files using Samtools mpileup command ("-uf" option), bcftools ("view -cg" option) and vcfutils.pl (vcf2fq command).

References:

Chaisson MJ, Tesler G. Mapping single molecule sequencing reads using basic local alignment with successive refinement (BLASR): application and theory. BMC Bioinformatics. 2012;13:238.

Li H, Handsaker B, Wysoker A, et al. The Sequence Alignment/Map format and SAMtools. Bioinformatics. 2009;25:2078-2079.
