# Supplementary material for: Exonization of an Intronic LINE-1 Element Causing Becker Muscular Dystrophy as a Novel Mutational Mechanism in Dystrophin Gene
Source: Genes (Basel). 2017 Oct 3;8(10):253. doi: 10.3390/genes8100253 (PMC5664103; doi:10.3390/genes8100253)
Supplement: Supplementary file 1 [file genes-08-00253-s001.zip › Data S2.pdf]

Data S2. LINE-1 sequence

> LINE-1_DMD_int51

```
   1 ggaggaggag ccaagatggc cgaataggaa cagctccggt ctacagctcc cagcgtgagc
  61 gacgcagaag acgggtgatt tctgcatttc catctgaggt accgggttca tctcaatagg
 121 gagtgccaga cagtgggcgc aggccagtgt gtgtgcgcac cgtgcgcaag ccgaagcagg
 181 gcgaggcatt gcctcacctg ggaagcgcaa ggggtcaggg agttcccttt ccgagtcaaa
 241 gaaaggggtg acggacgtac ctggaaaatc gggtcactcc cacccgaata ttgcgctttt
 301 cagaccggct taagaaacgg cgcaccacga gactatatcc cacacctggc tcagagggtc
 361 ctacgcccac ggaatctcgc tgattgctag cacagcagtc tgagatcaaa ctgcaaggcg
 421 gcaacgaggc tgggggaggg gcgcccgcca ttgcccaggc ttgcttaggt aaacaaagca
 481 gccgggaagc tcgaactggg tggagcccac cacagctcaa ggaggcctgc ctgcctctgt
 541 aggctccacc tctgggggca gggcacagac aaacaaaaag acagcagtaa cctctgcaga
 601 cttaagtgtc cctgtctgac agctttgaag agagcagtgg ttctcccagc acgcagctgg
 661 agatctgaga acgggcagac tgcctcctca agtgggtccc tgacccctga cccccgagca
 721 gcctaactgg gaggcacccc ccagcagggg cacactgaca cctcacacgg cagggtattc
 781 caacagacct gcagctgagg gtcctgtctg ttagaaggaa aactaacaac cagaaaggac
 841 atctacaccg aaaacccatc tgtacatcac catcatcaaa gaccaaaagt agataaaacc
 901 acaaagatgg ggaaaaaaca gaacagaaaa actggaaact ctaaaacgca gagcgcctct
 961 cctcctccaa aggaacgcag ttcctcacta gcaacagaac aaagctggat ggagaatgat
1021 tttgacgagc tgagagaaga aggcttcaga cgatcaaatt actctgagct acgggaggac
1081 attcaaacca aaggcaaaga agttgaaaac tttgaaaaaa atttagaaga atgtataact
1141 agaataacca atacagagaa gtgcttaaag gagctgatgg agctgaaaac caaggctcga
1201 gaactacgtg aagaatgcag aagcctcagg agccgatgcg atcaactgga agaaagggta
1261 tcagcaatgg aagatgaaat gaatgaaatg aagcgagaag ggaagtttag agaaaaaaga
1321 ataaaaagaa atgagcaaag cctccaagaa atatgggact atgtgaaaag accaaatcta
1381 cgtctgattg gtgtacctga aagtgatgtg gagaatggaa ccaagttgga aaacactctg
1441 caggatatta tccaggagaa cttccccaat ctagcaaggc aggccaacgt tcagattcag
1501 gaaatacaga gaacgccaca aagatactcc tcgagaagag caactccaag acacatactt
1561 gtcagattca ccaaagttga aatgaaggaa aaaatgttaa gggcagccag agagaaaggt
1621 caggttaccc tcaaaggaaa gcccatcaga ctaacagcgg atctctcggc agaaacccta
1681 caagccagaa gagagtgggg gccaatattc aacattctta aagaaaagaa ttttcaaccc
1741 agaatttcat atccagccaa actaagcttc ataagtgaag gagaaataaa atactttata
1801 gacaagcaaa tgctgagaga ttttgtcacc accaggcctg ccctaaaaga gctcctgaag
1861 gaatcactaa acatggaaag gaacaaccgg taccagccgc tgcaaaatca tgccaaaatg
1921 taaagacatc gagactagga agaaactgca tcaactaatg agcaaaatca ccagctaaca
```

1981 tcataatgac aggatcaaat tcacacataa caatattaac tttaaatata aatggactaa
2041 attctgcaat taaaagacac agactggcaa gttggataaa gagtcaagac ccatcagtgt
2101 gctgtattca ggaaacccat ctcacgtgca gagacacaca taggctcaaa ataaaaggat
2161 ggaggaagat ctaccaagca aatggaaaac aaaaaaaggc aggggttgca atcctagtct
2221 ctgataaaac agacttcaaa ccaacgaaga tcaaaagaga caaagaaggc cattacataa
2281 tggtaaaggg atcaattcaa caagaggagc taactatcct aaatatttat gcacccaata
2341 caggagcacc cagattcata aagcaagtcc tcagtgagct acaaagagac ttagactccc
2401 acacattaat aatgggagac tttaacaccc cactgtcaac attagacaga tcaacgagac
2461 agaaagtcaa caaggatacc caggaattga actcagctct gcaccaagca gacctaatag
2521 acatctacag aactctccac cccaaatcaa cagaatatac attttttca gcaccacacc
2581 acacctattc caaaattgac cacatagttg gaagtaaagc tctcctcagc aaatgtaaaa
2641 gaacagaaat tataacaaac tatctctcag accacagtgc aatcaaacta gaactcagga
2701 ttaagaatct cactcaaagc cgctcaacta catggaaact gaacaacctg ctcctgaatg
2761 actactgggt acataacgaa atgaaggcag aaataaagat gttctttgaa accaacgaga
2821 acaaagacac cacataccag aatctctggg acgcattcaa agcagtgtgt agagggaaat
2881 ttatagcact aaatgcctac aagagaaagc aggaaagatc caaaattgac accctaacat
2941 cacaattaaa agaactagaa aagcaagagc aaacacattc aaaagctagc agaaggcaag
3001 aaataactaa aatcagagca gaactgaagg aaatagagac acaaaaaacc cttcaaaaaa
3061 tcaatgaatc caggagctgg ttttttgaaa ggatcaacaa aattgataga ccgctagcaa
3121 gactaataaa gaaaaaaaga gagaagaatc aaatagacac aataaaaaat gataaagggg
3181 atatcaccac cgatcctaca gaaatacaaa ctaccatcag agaatactac aaacacctct
3241 atgcaaataa actagaaaat ctagaagaaa tggatacatt cctcgacaca tacactctcc
3301 caagactaaa ccaggaagaa gttgaatctc tgaatagacc aataacaggc tctgaaattg
3361 tggcaataat caatagttta ccaaccaaaa agagtccagg accagatgga ttcacagccg
3421 aattctacca gaggtacaag gaggaactgg taccattcct tctgaaacta ttccaatcaa
3481 tagaaaaaga gggaatcctc cctaactcat tttatgaggc cagcatcatt ctgataccaa
3541 agccgggcag agacacaacc aaaaaagaga attttagacc aatatccttg atgaacattg
3601 atgcaaaaat cctcaataaa atactggcaa accgaatcca gcagcacatc aaaaagctta
3661 tccaccatga tcaagtgggc ttcatccctg ggatgcaagg ctggttcaat atacgcaaat
3721 caataaatgt aatccagcat ataaacagag ccaaagacaa aaaccacatg attatctcaa
3781 tagatgcaga aaaagccttt gacaaaattc aacaaccctt catgctaaaa actctcaata
3841 aattaggtat tgatgggacg tatttcaaaa taataagagc tatctatgac aaacccacag
3901 ccaatatcat actgaatggg caaaaactgg aagcattccc tttgaaaact ggcacaagac
3961 agggatgccc tctctcaccg ctcctattca acatagtgtt ggaagttctg gccagggcaa
4021 tcaggcagga gaaggaaata aagggtattc aattaggaaa agaggaagtc aaattgtccc
4081 tgtttgcaga cgacatgatt gtttatctag aaaaccccat catctcagcc caaaatctcc
4141 ttaagctgat aagcaacttc agcaaagtct caggatacaa aatcaatgta caaaaatcac

4201 aagcattctt atacaccaac aacagacaaa cagagagcca aatcatgggt gaactcccat
4261 tcacaattgc ttcaaagaga ataaaatact taggaatcca acttacaagg gatgtgaagg
4321 acctcttcaa ggagaactac aaaccactgc tcaaggaaat aaaagaggac acaaacaaat
4381 ggaagaacat tccatgctca tgggtaggaa gaatcaatat cgtgaaaatg gccatactgc
4441 ccaaggtaat ttacagattc aatgccatcc ccatcaagct accaatgact ttcttcacag
4501 aattggaaaa aactacttta aagttcatat ggaaccaaaa aagagcccgc attgccaagt
4561 caatcctaag ccaaaagaac aaagctggag gcatcacact acctgacttc aaactatact
4621 acaaggctac agtaaccaaa acagcatggt actggtacca aaacagagat atagatcaat
4681 ggaacagaac agagccctca gaaataatgc cgcatatcta caactatctg atctttgaca
4741 aacctgagaa aaacaagcaa tggggaaagg attccctatt taataaatgg tgctgggaaa
4801 actggctagc catatgtaga aagctgaaac tggatccctt ccttacacct tatacaaaaa
4861 tcaattcaag atggattaaa gacttaaatg ttagacctaa aaccataaaa accctagagg
4921 aaaacctagg cattaccatt caggacatag gcgtgggcaa ggacttcatg tccaaaacac
4981 caaaagcaat ggcaacaaaa accaaaattg acaaatggga tctaattaaa ctaaagagct
5041 tttgcacagc aaaagaaact accatcagag tgaacaggca acctacaaca tgggagaaaa
5101 tttttgcaac ctactcatct gacaaagggc taatatccag aatctacaat gaactcaaac
5161 aaatttacaa gaaaaaaaca aacaacccca tcaaaaagtg ggcgaaggac atgaacagac
5221 acttctcaaa agaagacatt tatgcagcca aaaaacacat gaaaaaatgc tcatcatcac
5281 tggccatcag agaaatgcaa atcaaaacca ctatgagata tcatctcaca ccagttagaa
5341 tggcaatcat taaaaagtca ggaaacaaca ggtgctggag aggatgtgga gaaataggaa
5401 cacttttaca ctgttggtgg gactgtaaac tagttcaacc attgtggaag tcagtgtggc
5461 gattcctcag ggatctagaa ctagaaatac catttgaccc agccatccca ttactgggta
5521 tatacccaaa ggactataaa tcatgctgct ataaagacac atgcacacgt atgtttattg
5581 cggcactatt cacaatagca aagacttgga accaacccaa atgtccaaca atgatagtct
5641 ggattaagaa aatgtggcac atatacacca tggaatacta tgcagccata aaaaatgatg
5701 agttcatatc ctttgtaggg acatggatga aattggaaac catcattctc agtaaactat
5761 cgcaagaaca aaaaaccaaa cactgcatat tctcactcat aggtgggaat tgaacaatga
5821 gatcacatgg acacaggaag gggaatatca cactctgggg actgtggtgg ggtcggggga
5881 gggggaggg atagcattgg gagatatacc taatgctaga tgacacatta gtgggtgcag
5941 tgcaccagca tggcacatgt atacatatgt aactaacctg cacaatgtgc acatgtaccc
6001 taaaactgag agtataataa aaaaaaaaaa ataaaaaata aaaaaaaaaa gaaaaaaaaa
6061 aaaaaaaaaa aaaaaaaaaa aaaaaaaaaa aaaaaa
