# Supplementary material for: Exonization of an Intronic LINE-1 Element Causing Becker Muscular Dystrophy as a Novel Mutational Mechanism in Dystrophin Gene
Source: Genes (Basel). 2017 Oct 3;8(10):253. doi: 10.3390/genes8100253 (PMC5664103; doi:10.3390/genes8100253)
Supplement: Supplementary file 1 [file genes-08-00253-s001.zip › Figure S1.pdf]

Figure S1. Bioinformatic splice-site and branch-point analysis. To identify candidate regions for the LINE-1 insertion, different algorithms were used to evaluate potential acceptor splice sites and branch points within intron 51, namely the SpliceSiteFinder-Like, MaxEntScan (Yeo et al., 2004), NNSPLICE (Reese et al., 1997), GeneSplicer (Pertea et al., 2001) and Human Splice Finder (Desmet et al., 2009), integrated in Alamut Visual software (v2.8, Interactive Biosoftware, Rouen, France).

REGION 1 – Potential acceptor splice-site

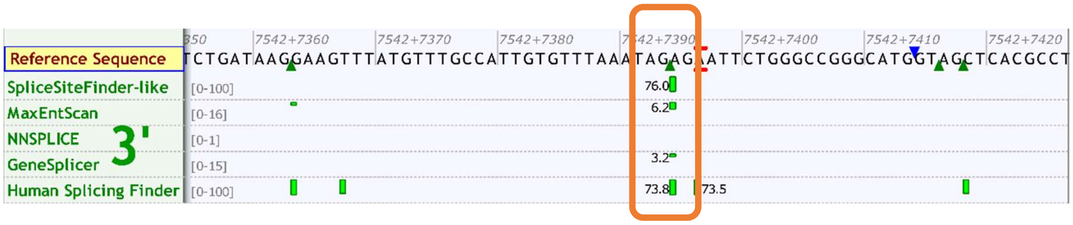

REGION 1 – Potential branch-points

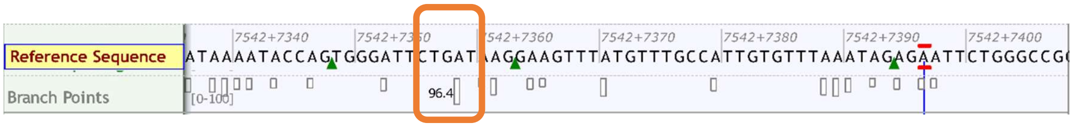

REGION 2 – Potential acceptor splice-site

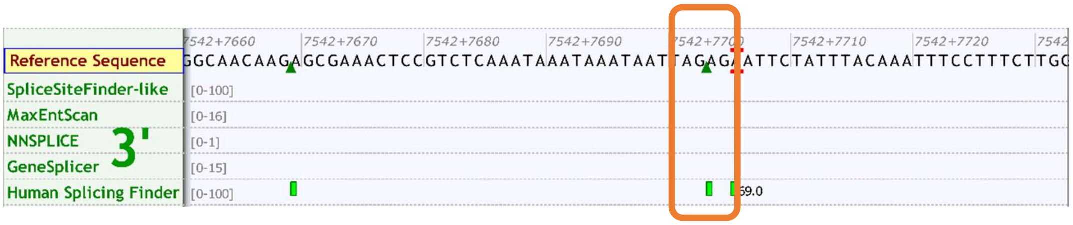

REGION 2 – Potential branch-points

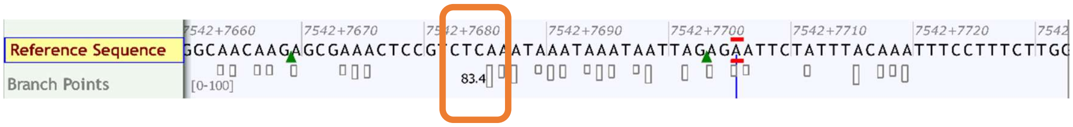

REGION 3 – Potential acceptor splice-site

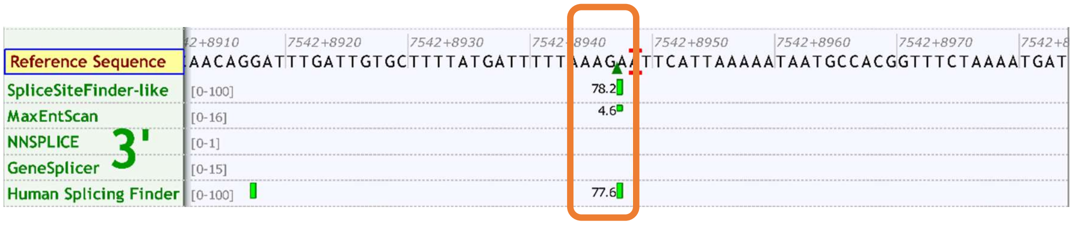

REGION 3 – Potential branch-points

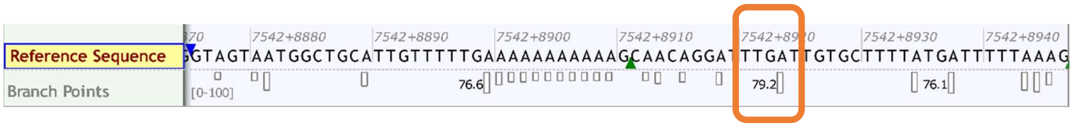

REGION 4 – Potential acceptor splice-site

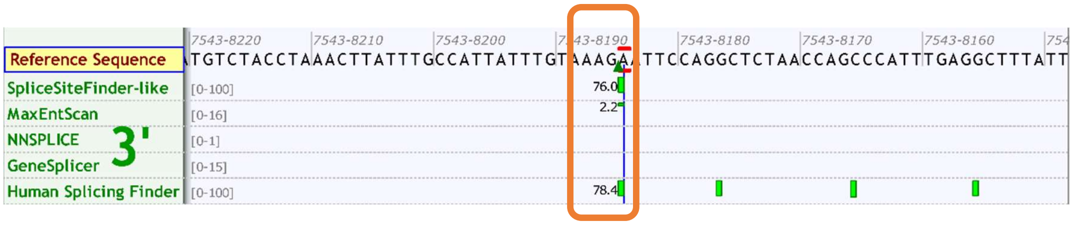

REGION 4 – Potential branch-points

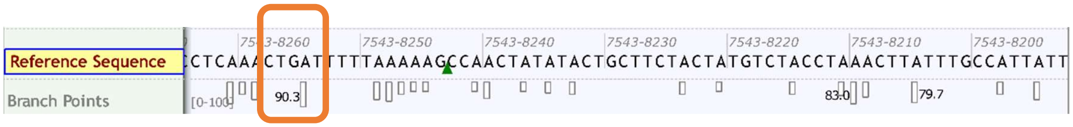

REGION 5 – Potential acceptor splice-site

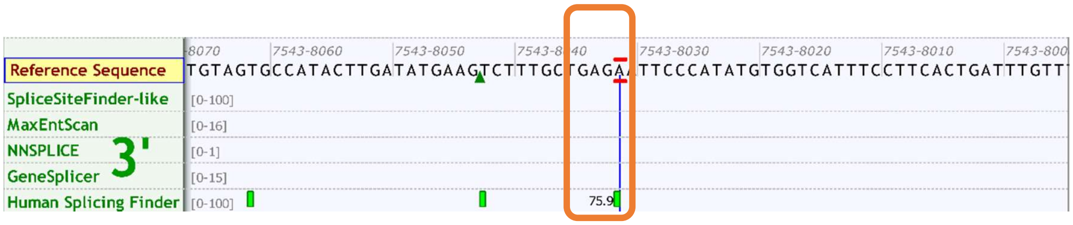

REGION 5 – Potential branch-points

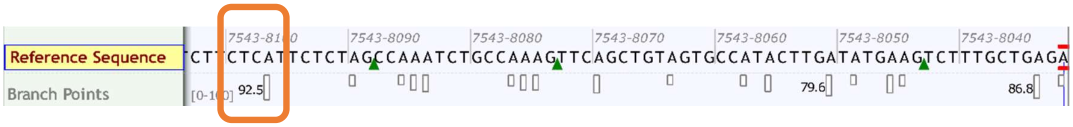

References:

Desmet FO, Hamroun D, Lalande M, Collod-Béroud G, Claustres M, Béroud C. Human Splicing Finder: an online bioinformatics tool to predict splicing signals. Nucleic Acids Res. 2009;37(9):e67.

Pertea M, Lin X, Salzberg SL. GeneSplicer: a new computational method for splice site prediction. Nucleic Acids Res. 2001;29(5):1185-90.

Reese MG, Eeckman FH, Kulp D, Haussler D. Improved splice site detection in Genie. J Comput Biol. 1997;4(3):311-23.

Yeo G, Burge CB. Maximum entropy modeling of short sequence motifs with applications to RNA splicing signals. J Comput Biol. 2004;11(2-3):377-94.
