# Supplementary material for: Exonization of an Intronic LINE-1 Element Causing Becker Muscular Dystrophy as a Novel Mutational Mechanism in Dystrophin Gene
Source: Genes (Basel). 2017 Oct 3;8(10):253. doi: 10.3390/genes8100253 (PMC5664103; doi:10.3390/genes8100253)
Supplement: Supplementary file 1 [file genes-08-00253-s001.zip › Figure S2.pdf]

Figure S2. Homology search bioinformatics. The search for the origin of the 103 bp sequence identified in the patient's DMD transcripts resorted to the Basic Local Alignment Search Tool (BLAST v2.2.32, http://blast.ncbi.nlm.nih.gov/) at the nucleotide level.

A)

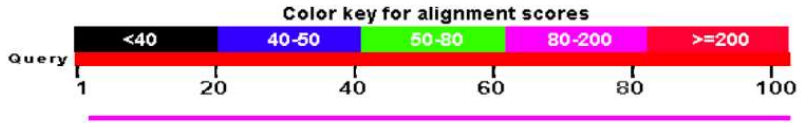

B)

Download GenBank Graphics

Human L1 element L1.21 p40 and putative p150 genes, complete cds

Sequence ID: gb|U93570.1|HSU93570 **Length:** 6020 **Number of Matches:** 1

Range 1: 1 to 98 GenBank Graphics ▼ Next Match ▲ Previous Match

Score	Expect	Identities	Gaps	Strand
182 bits(98)	1e-42	98/98(100%)	0/98(0%)	Plus/Plus

```
Query  6     GGAGGAGGAGCCAAGATGGCCGAATAGGAACAGCTCCGGTCTACAGCTCCCAGCGTGAGC  65
             ||||||||||||||||||||||||||||||||||||||||||||||||||||||||||||
Sbjct  1     GGAGGAGGAGCCAAGATGGCCGAATAGGAACAGCTCCGGTCTACAGCTCCCAGCGTGAGC  60

Query  66    GACGCAGAAGACGGGTGATTTCTGCATTTCCATCTGAG  103
             ||||||||||||||||||||||||||||||||||||||
Sbjct  61    GACGCAGAAGACGGGTGATTTCTGCATTTCCATCTGAG  98
```

C)

Download GenBank Graphics

Human L1 element L1.14 p40 gene, complete cds

Sequence ID: gb|U93566.1|HSU93566 **Length:** 6020 **Number of Matches:** 1

Range 1: 1 to 98 GenBank Graphics ▼ Next Match ▲ Previous Match

Score	Expect	Identities	Gaps	Strand
182 bits(98)	1e-42	98/98(100%)	0/98(0%)	Plus/Plus

```
Query  6     GGAGGAGGAGCCAAGATGGCCGAATAGGAACAGCTCCGGTCTACAGCTCCCAGCGTGAGC  65
             ||||||||||||||||||||||||||||||||||||||||||||||||||||||||||||
Sbjct  1     GGAGGAGGAGCCAAGATGGCCGAATAGGAACAGCTCCGGTCTACAGCTCCCAGCGTGAGC  60

Query  66    GACGCAGAAGACGGGTGATTTCTGCATTTCCATCTGAG  103
             ||||||||||||||||||||||||||||||||||||||
Sbjct  61    GACGCAGAAGACGGGTGATTTCTGCATTTCCATCTGAG  98
```

D)

Local Alignments*

Name	From	To	Name	From	To	Dir	Sim	Pos/Mm:Ts	Score
9978.tmp/data.ori	1	98	L1HS	2	99	d	1.0000	99.0000	938

```
1  GGAGGAGGAGCCAAGATGGCCGAATAGGAACAGCTCCGGTCTACAGCTCCCAGCGTGAGCGACGCAGAAG 70
   ||||||||||||||||||||||||||||||||||||||||||||||||||||||||||||||||||||||
2  GGAGGAGGAGCCAAGATGGCCGAATAGGAACAGCTCCGGTCTACAGCTCCCAGCGTGAGCGACGCAGAAG 71

71 ACGGGTGATTTCTGCATTTCCATCTGAG 98
   ||||||||||||||||||||||||||||
72 ACGGGTGATTTCTGCATTTCCATCTGAG 99
```

Legend: A) BLAST results showing that a 95.1% homology (98/103) was obtained against two human LINE-1 sequences: B) L1.21 (sequence ID: U93570) and C) L1.14 (sequence ID: U93566). D) To confirm the nature of this sequence, analysis of repetitive elements was performed using CENSOR software (http://www.girinst.org/censor/). Censor results showing 100% similarity with a stretch of 98 nucleotides belonging to the consensus sequence of the L1Hs family.
