# Supplementary material for: Exonization of an Intronic LINE-1 Element Causing Becker Muscular Dystrophy as a Novel Mutational Mechanism in Dystrophin Gene
Source: Genes (Basel). 2017 Oct 3;8(10):253. doi: 10.3390/genes8100253 (PMC5664103; doi:10.3390/genes8100253)
Supplement: Supplementary file 1 [file genes-08-00253-s001.zip › Figure S3.pdf]

Figure S3. Southern-blot analysis

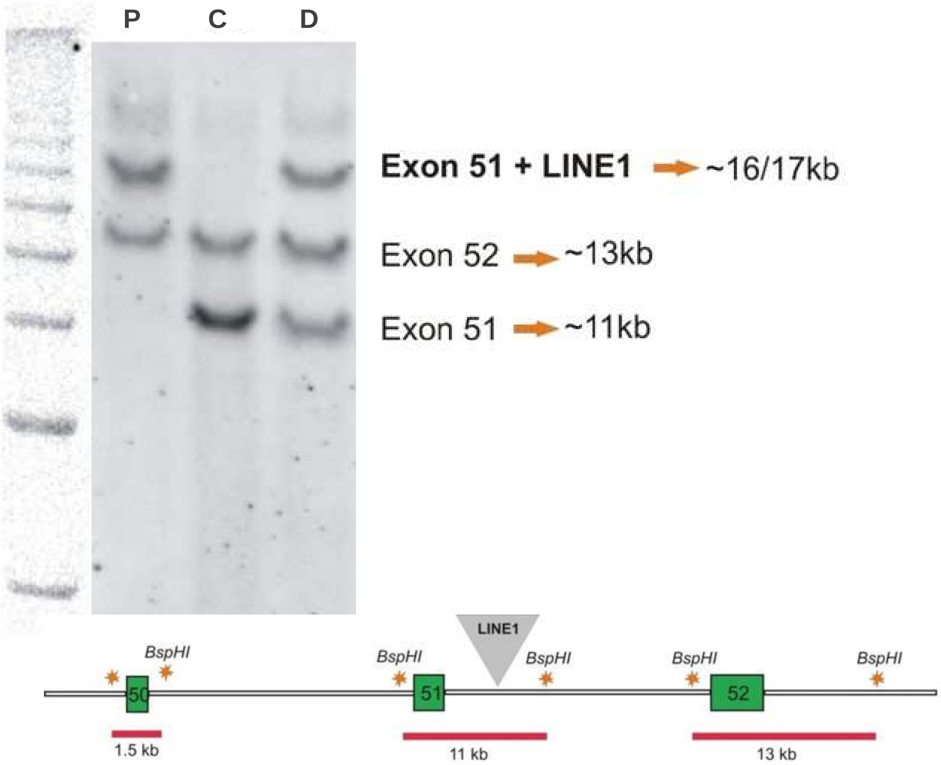

Legend: Southern blot and hybridization analysis using a cDNA probe recognizing exons 50 to 52. A larger fragment (increment of ~ 6 Kb) was identified in the patient (P) and his daughter (D), and not detected in the control sample (C).

Material & Methods:

Genomic DNA (gDNA) samples from patient, daughter, as well as male normal control, were digested with BspHI (NewEngland Biolabs, Beverly, MA), resolved on a 0.7% agarose gel and vacuum transferred to a GeneScreen Plus membrane (Perkin Elmer, Waltham, MA) using an saline method. A custom cDNA probe recognizing exons 50-52 was prepared using the digoxigenin (DIG) DNA Labeling Kit (Roche Applied Science, Indianapolis, IN). The membrane was incubated with this probe overnight using the Easy Hyb Buffer (Roche Applied Science). Subsequently, the membrane was washed at 60ºC in 1xSSC (saline-sodium citrate) /0.1% SDS (Sodium dodecyl sulfate) and 0.5xSSC/0.1% SDS, and prepared with DIG Wash and Block Buffer

Set (Roche Applied Science, Indianapolis, IN). Incubation was performed with Anti-DIG-AP conjugate (Roche Applied Science, Indianapolis, IN), and the DIG-labeled probe was detected with ready-to-use CDP-Star (Roche Applied Science, Indianapolis, IN).
